# Therapeutic Effects of Wenxin Keli in Cardiovascular Diseases: An Experimental and Mechanism Overview

**DOI:** 10.3389/fphar.2018.01005

**Published:** 2018-09-05

**Authors:** Guihua Tian, Yang Sun, Shuo Liu, Chengyu Li, Shiqi Chen, Ruijin Qiu, Xiaoyu Zhang, Youping Li, Min Li, Hongcai Shang

**Affiliations:** ^1^Chinese Cochrane Center, West China Hospital, Sichuan University, Chengdu, China; ^2^Key Laboratory of Chinese Internal Medicine of Ministry of Education and Beijing, Dongzhimen Hospital, Beijing University of Chinese Medicine, Beijing, China; ^3^Institute of Integration of Traditional Chinese and Western Medicine, Guangzhou Medical University, Guangzhou, China

**Keywords:** Wenxin Keli, WXKL, cardioprotective effects, antiarrhythmic effects, mechanism

## Abstract

Cardiovascular diseases (CVDs) are the major public health problem and a leading cause of morbidity and mortality on a global basis. Wenxin Keli (WXKL), a formally classical Chinese patent medicine with obvious efficacy and favorable safety, plays a great role in the management of patients with CVDs. Accumulating evidence from various animal and cell studies has showed that WXKL could protect myocardium and anti-arrhythmia against CVDs. WXKL exhibited its cardioprotective roles by inhibiting inflammatory reaction, decreasing oxidative stress, regulating vasomotor disorders, lowering cell apoptosis, and protection against endothelial injure, myocardial ischemia, cardiac fibrosis, and cardiac hypertrophy. Besides, WXKL could effectively shorten the QRS and Q-T intervals, decrease the incidence of atrial/ventricular fibrillation and the number of ventricular tachycardia episodes, improve the severity of arrhythmias by regulating various ion channels with different potencies, mainly comprising peak sodium current (I_Na_), late sodium current (I_NaL_), transient outward potassium current (I_to_), L-type calcium current (I_CaL_), and pacemaker current (I_f_).

## Introduction

Cardiovascular diseases (CVDs) are the major public health problem and a leading cause of premature death throughout the world. Approximately 17.7 million people died from CVDs in 2015, accounting for 31% of all global deaths (WHO, 2017). As a result of population growth, the aging of populations, and epidemiologic changes in disease, the prevalence of cardiovascular morbidity and mortality continues to rise (Roth et al., 2015). Cardiac arrhythmias, a type of CVDs, is a disease that is caused by abnormal electrical activity in the heart rate (HR) or rhythm, further affecting the pumping function of the heart (Nattel et al., 2014). Currently, the electrophysiological mechanisms responsible for cardiac arrhythmias mainly divided into two aspects: enhanced or abnormal impulse formation (i.e., focal activity) and conduction disturbances (i.e., re-entry) (Antzelevitch and Burashnikov, 2011), which results in premature beat, atrial fibrillation (AF), ventricular fibrillation (VF), atrioventricular block, and other arrhythmic diseases.

It is estimated that the number of patients with AF in 2030 in Europe will be 14–17 million and the number of new cases of AF per year at 120,000–215,000 (Zoni-Berisso et al., 2014). Around 40–50% of all cardiovascular deaths are sudden cardiac deaths, while approximately 80% of these are caused by ventricular tachyarrhythmias (Mehra, 2007). In addition, cardiac arrhythmia is also caused by cardiovascular organic lesions, such as myocardial ischemia, fibrosis, cardiac hypertrophy, impaired cardiac function and so forth, which further influence electrophysiological activity by changing the structure of the heart (Nguyen et al., 2017; Wu et al., 2017a). Traditional Chinese medicine (TCM) has more than 2,000 years of history and has gained widespread clinical applications in patients with CVDs for thousands of years and is still being commonly used in modern times in both China and elsewhere worldwide (Hao et al., 2017).

Wenxin Keli (WXKL), a formally classical Chinese patent medicine developed at the Guang’anmen Hospital of the Chinese Academy of Chinese Medical Sciences, is the first Chinese anti-arrhythmic medicine to be approved by the China Food and Drug Administration and has been increasingly used as an alternative approach for CVDs globally. The main ingredients of WXKL consist of *Codonopsis Radix (Dang Shen), Polygonati Rhizoma (Huang Jing), Notoginseng Radix Et Rhizoma (San Qi), Ambrum (Hu Po), and Nardostachyos Radix Et Rhizoma (Gan Song)*, which can tonify qi, supply yin, promote blood circulation and remove blood stasis according to the TCM theory (Pharmacopoeia, 2015). Detailed extraction process and drug instruction were shown in **Supplementary Material 1**. Several studies exhibited that WXKL had better clinical efficacy in the treatment of CVDs (Wang et al., 2016). WXKL appeared to be more effective in improving P-wave dispersion as well as maintenance of sinus rhythm in patients with paroxysmal AF (Chen et al., 2013b). WXKL also reduced the frequency of ventricular premature complexes (VPCs), increased left ventricular ejection fraction (LVEF) and 6-min walking test in patients with VPCs and heart failure (HF) (Chen et al., 2014; He et al., 2016; Li et al., 2017a). Similarly, many studies demonstrated the key role of WXKL against CVDs in animal and cell experiments, but there is a lack of comprehensive and systematic evidence. This review summarizes extensively current experimental and mechanism studies on the use of WXKL in CVDs, and our understanding of WXKL’s cardioprotective and antiarrhythmic effects.

## Methodology

The terms “Wenxin” or “Wen xin” were searched as “Title/Abstract” and MeSH terms in PubMed, China National Knowledge Infrastructure(CNKI) and SinoMed database. Articles related to therapeutic effects in CVDs were picked out manually. All articles with abstract were included and no language restrictions was applied.

## Results

### The Cardioprotective Effects of WXKL

**FIGURE 1:**
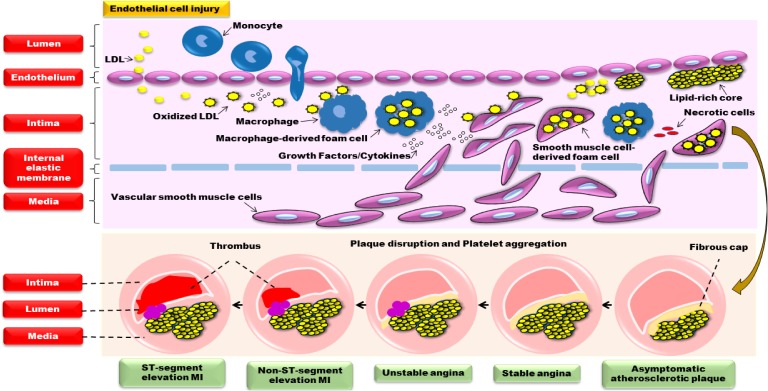
The pathological mechanism of atherosclerosis in CVDs.

The damage of endothelial cell structure and function is the main pathological basis of many CVDs (Hadi et al., 2005; Gutiérrez et al., 2013). When stimulated by various factors such as dyslipidemia immune, oxidative stress and virus, the endothelium will be injured. Masses of low density lipoprotein (LDL) enters intima and is converted into oxidized LDL (ox-LDL), which will lead to the release of cytokines (interleukin-1, IL-1; interleukin-6, IL-6; monocyte chemoattractant protein-1, MCP-1) and monocyte emigration into intima. When monocytes enter the intima through the endothelium, they will become macrophages and provide antigens for the lymphocytes, producing a local immune response. Macrophages consume a substantial number of ox-LDL and convert them into macrophage-derived foam cells, which release large amounts of cytokines and growth factors, resulting in the migration and proliferation of smooth muscle cells from media to intima. And smooth muscle cells further uptake lipid then become smooth muscle cell-derived foam cells. Those foam cells accumulate into masses, forming pale-yellow lipid plaques. With collagen fibrils proliferation, yellow-gray fibrous cap will cover intima surface, causing coronary atherosclerosis and angina. In addition, some immune cells, inflammatory factors and proteolytic enzymes easily weaken the thin fibrous cap and transform stable plaque to an unstable vulnerable plaque, contributing to plaque disruption, platelet aggregation and thrombosis, finally resulting in myocardial ischemia and necrosis, generating acute coronary syndrome (ACS) and sudden death (Santos-Gallego et al., 2014; Androulakis et al., 2017). Thus, the main pathological mechanism of atherosclerosis in CVDs is closely related to endothelial erosion, disorders of lipid metabolism, inflammatory reaction, myocardial apoptosis, and so forth (**Figure 1**).

Twenty-two articles exerted the cardioprotective properties of WXKL in CVDs, involving in myocardial ischemia, myocardial infarction (MI), ischemia/reperfusion (I/R), transverse aortic constriction (TAC), congestive heart failure, spontaneously hypertensive animal models and Ang II/Norepinephrine (NE)-induced H9C2 cells, the basic characteristic of included studies was shown in **Table 1**. And the detailed experimental mechanism was as follows.

**Table 1:** The cardioprotective effects of WXKL.

Experimental models	Outcome measures	Effects	Reference
TAC SD rats	FS↑, p-CaMK II↓, p-PLB↑, p-RYR2↑, Collagen↓, APD_90_↓	Decreasing cardiac hypertrophy and inhibited the arrhythmia	Yang et al., 2017a
TAC SD rats	Q-T dispersion↓, LVPWd↓, IVSd↓, LVIDs↓, Cx43 mRNA↑	Improving cardiac hypertrophy and remodeling of Cx43 in myocardium	Long et al., 2017
TAC rabbits	EF↑, FS↑, LVESD↓, LVEDD↓, LVESV↓, LVEDV↓, SERCA2a mRNA and protein↑	Improving cardiac function	Lian et al. (2014)
MI SD rats	LVEF↑, infarct size↓, Cx43-p↑, Cx43-np↓, miR-1↑, SRF↑, VFT↑	Protecting the ultrastructure of the gap junctions and inhibited the arrhythmia	Wu et al., 2017a,b
MI SD rats	EF↑, FS↑, LViDd↓, LViDs↓, ESV↓, EDV↓, apoptosis rate↓, Ang II↓	Improving the cardiac function, reversing ventricular remodeling and inhibiting myocardial apoptosis	Wu et al., 2013
MI SD rats	EF↑, FS↑, LViDd↓, LViDs↓, ESV↓, EDV↓, CaMKII↓, p-CaMKII↓, PLB↓, p-PLB↑, RYR2↑, FKBP12.6↑, incidences of EADs and DAD↓,	Improving the cardiac function and inhibiting the arrhythmia by regulating the CaMKII signal transduction pathway	Xing et al., 2013
MI SD rats	ET-1↓, NO↑	Protection of vascular endothelial cells	Guan et al., 2011
MI rabbits	EF↑, FS↑, ESD↓, EDD↓, ESV↓, EDV↓, collagen↓; apoptosis rate↓; CX3CR1↓, MRC1↓, FPR1↓, CTSC↓, TTC5↓, ACE↓, EDN1↓, RSPO3↑	Inhibiting inflammation, renin-angiotensin system, and myocardial apoptosis	Zheng et al., 2016
I/R SD rats	LVEF↑, LVFS↑ LVAWs↑, E/A↑, Peak vel↑, HR↑, LVSP↑, LVDP↑, +dp/dt_max_↑, -dp/dt_max_↓, taurine↓ acetoacetate↓	Modulating the key metabolites, overcoming the oxidative stress and the shortage of energy sources	Jiang et al., 2017
I/R SD rats	NO↑, SOD↑, MDA↓	Reducing free radicals and antioxidative stress	Wang et al., 2014
I/R SD rats	Myocardial infarction size↓	Protecting myocardium	Wang and Liu, 2014
I/R rabbits	MDA↓, LDH↓, CK↓, SOD↑, incidence of arrhythmia↓	Protecting myocardium and anti-arrhythmia	Zhou and Sun, 2015
Spontaneously hypertensive rats	Cx43-p↑	Improving myocardial remodeling and delaying myocardial fibrosis	Wu et al., 2017c
CHF Wistar rats	ET-1↓	Protection of vascular endothelial cells	Liu et al., 2007a
Beagle dogs	CK↓, CK-MB, LDH↓, serum globulin↑, serum lysozyme↑	Protection of myocardium	Zhang et al., 2012
ISO-induced SD rats	ST segment elevation _(MAX)_↓, CK↓	Protecting myocardium	Xiang et al., 2015
ISO-induced SD rats	β-catenin protein↓, c-myc↓	Improving cardiac hypertrophy	Wang et al., 2011a
ISO-induced SD rats	LVEDP↓, +dp/dt_max_↑	Improving cardiac function	Zhou et al., 2007
ADR-induced Wistar rats	LVSP↑, LVEDP↓, +dp/dt_max_↑, -dp/dt_max_↓, Ang II↓	Improving cardiac function	Liu et al., 2007b
Ang II-inducedH9C2 cells	Cell length and width↓	Improving cytoskeletal protein and anti-hypertrophy	Ren et al., 2016
NE-induced H9C2 cells	Cx43 mRNA↑, cell length and width↓, cell proliferation↑	Anti-hypertrophy	Yang et al., 2012

*SD, Sprague-Dawley; PLB, phospholamban; RYR2, Ryanodine receptor 2; SRF, serum response factor; LVAWs, left ventricular end-systolic anterior wall; E/A, E-wave to A-wave; Peak vel, aortic valve peak velocity; LVSP, left ventricular systolic pressure; LVDP, left ventricular development pressure; +dp/dt_*max*_, left ventricular maximum upstroke velocity; -dp/dt_*max*_, left ventricular maximum descent velocity; ESD, end-systolic dimension; EDD, end-diastolic dimension; ESV, end-systolic volume; EDV, end-diastolic volume; EDN1, endothelin 1; FKBP12.6, FK binding protein 12.6; SHR, spontaneously hypertensive rats; LVPWd, left ventricular posterior wall end-diastolic thickness; IVSd, interventricular septum thickness at end-diastole; CHF, congestive heart failure; LVEDP, left ventricular end diastolic pressure*.

#### Anti-inflammation

**FIGURE 2:**
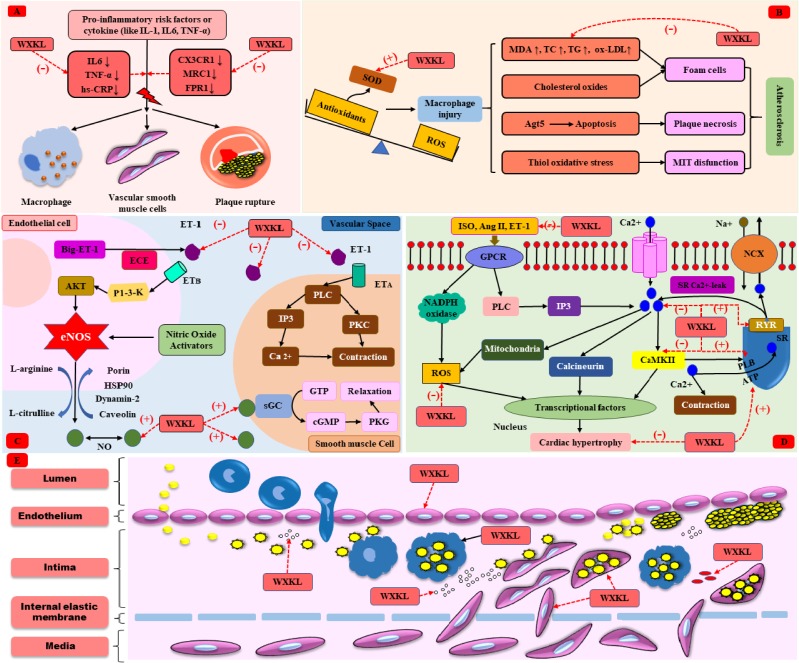
The cardioprotective mechanism of WXKL. **(A)** Anti-inflammation. **(B)** Anti-oxidative stress. **(C)** Regulation of vasomotor. **(D)** Anti-cardiac hypertrophy. **(E)** Anti-atherosclerosis.

Inflammation dominates in atherosclerosis and CVDs. Immune cells gather in the early atherosclerotic lesions and induce migration and proliferation of smooth muscle cells. The aggravation of inflammation in the arterial wall also causes instability of atheromatous plaques and formation of occlusive thrombosis, contributing to atherosclerotic CVD events, including ACS and stroke (Ohira et al., 2017). WXKL exhibited its anti-inflammation property by lowering the level of IL-6, tumor necrosis factor-α (TNF-α) and high sensitivity C-reactive protein (hs-CRP) (Cao et al., 2016). In addition, WXKL could downregulate inflammatory related gene expression, such as chemokine receptor 1 (CX3CR1), mannose receptor ctype1 (MRC1), and formyl peptide receptor 1 (FPR1) (Zheng et al., 2016), as shown in **Figure 2A**.

#### Anti-oxidative Stress

Oxidative stress is defined as an imbalance between the generation of reactive oxygen species (ROS) and the ability to scavenge these ROS by endogenous antioxidative systems, where ROS overwhelms the antioxidative capacity. Oxidative stress enables to result in irreversible damage to cell membranes, DNA and cellular metabolism, and plays a crucial role in the pathogenesis of CVDs (Farías et al., 2017). As far as we know, superoxide dismutase (SOD) is a well-known, first-line defiance antioxidative enzyme that protects cells from the toxic effects of superoxide radicals, while malondialdehyde (MDA) is the final product of lipid peroxidation induced by ROS, which can result in cross-linking in lipids, proteins, and nucleic acids, and is frequently used to define oxidative stress (Bulut et al., 2007; Valko et al., 2016). Oxidative stress mediated atherosclerosis and causes CVDs (Yang et al., 2017b). Recent articles indicated that MDA was slightly increased and SOD was slightly decreased in the I/R rats/rabbits when compared with the controls, and WXKL could recovery the level of SOD and MDA, play a crucial role in anti-oxidative stress (Wang et al., 2014; Zhou and Sun, 2015). Moreover, WXKL could improve the secretions of taurine and ketone bodies to overcome the oxidative stress and the shortage of energy sources induced by I/R (Jiang et al., 2017). The mechanism of atherosclerosis caused by oxidative stress (Yang et al., 2017b) and the anti-oxidative role of WXKL were shown in **Figure 2B**.

#### Endothelial Protective Effect

The endothelium is a monolayer of cells covering the internal lumen of all blood vessels, thereby separating the blood from the vascular wall and organ tissues. Dysfunction of endothelial cells is directly associated with impaired vasorelaxation, increased inflammation, and increased migration and proliferation of smooth muscle cells, is the initiating factor in CVDs. Thus, protecting VECs from injury is very significant. In ischemic myocardial tissue, the nucleus of vascular endothelial cells was irregular, the chromatin agglomerated, the basement membrane was dissolved, the endothelia cells appeared protuberance, the cytoplasm was dense, and the pericytes were edema and cavitation. The mitochondria were slightly swollen in the endothelial cells. Current animal evidence indicated that WXKL enabled to effectively recovery ultrastructure of damaged endothelial cells in a dose-depended way (Liu et al., 2007a; Guan et al., 2011; Wang et al., 2014). Besides, anti-inflammatory and anti-oxidative effects were also beneficial to protect endothelium.

#### Regulation of Vasomotor

Nitric oxide (NO) is a multifunctional versatile molecule, especially regulating vascular tone. In the vasculature, NO directly activates the heme moiety of soluble guanylate cyclase leading to the production of cyclic GMP (cGMP). An increase in intracellular cGMP further result in the activation of protein kinase G (PKG), finally contributing to vasodilatation. Endothelin-1 (ET-1) is also a kind of endothelium-derived mediator, mediating vasoconstriction by activating PLC-induced endothelin signaling pathway. The delicate equilibrium between NO and ET-1 is essential for maintaining vascular homeostasis (Khimji and Rockey, 2010). A lot of results indicate that plasma ET-1 levels are upregulated in patients with coronary heart disease (Hoffmann et al., 1998; Zhu et al., 2011). In addition, many studies in our review showed that NO decreased and ET-1 increased in MI or I/R rats. And WXKL could restore this imbalance between NO and ET-1 (Liu et al., 2007a; Guan et al., 2011; Wang et al., 2014). The role of NO and ET-1 in regulating vasomotor (Khimji and Rockey, 2010) and effects of WXKL were shown in **Figure 2C**.

### Anti-myocardial Ischemia

Myocardial ischemia refers to the decrease in the blood perfusion of the heart, leading to the reduction of oxygen supply to the heart and the abnormal metabolism of the myocardial energy. Minutes after the onset of ischemia, reversible ultrastructural cardiomyocyte changes appear, including cellular and mitochondrial swelling and glycogen depletion, causing irreversible myocardial necrosis. MI defined as the death of cardiac myocytes due to prolonged ischemia, affecting millions of individuals each year and represents a considerable economic burden to healthcare systems worldwide (Tao et al., 2017). When AMI occurs, reperfusion of the ischemic myocardium is a valuable approach for limiting infarct size. However, reperfusion itself paradoxically result in further complications involving acceleration of cell death, diminished contractile function (stunning), and arrhythmias, which is called myocardial I/R injury. No effective therapy is currently available to protect the heart from this I/R injury (Eltzschig and Eckle, 2011).

In isoproterenol (ISO) or adriamycin hydrochloride (ADR) induced myocardial ischemia animal model, WXKL enabled to decrease the maximal value of ST segment elevation and the level of creatine kinase (CK), improve the systolic and diastolic function of left ventricle and prevented left ventricular dilatation (Liu et al., 2007b; Zhou et al., 2007; Xiang et al., 2015). Underwent the surgery of ligation of the left anterior descending coronary artery, MI rats or rabbits inhibited that myocardial fibers arrangement was discorded, numerous neutrophil granulocytes were seen to be infiltrating, and wide range of necrosis observed, while some cytoplasts showed intense staining according to myocardial histopathology. In addition, there was a large number of myocardial apoptotic nuclei and the apoptosis rates were significantly increased after the coronary artery occlusion surgery. WXKL effectively restored the LVEF and left ventricular fractional shortening (LVFS), improved left ventricular end-diastolic dimension (LViDd) and left ventricular end-systolic dimension (LViDs), reduced MI size and apoptosis rate (Zeng et al., 2006; Liu et al., 2012; Guo et al., 2013; Wu et al., 2013, 2017a,b; Xing et al., 2013; Wang and Liu, 2014; Zheng et al., 2016; Jiang et al., 2017). And WXKL also could downregulate genes associated with apoptosis, such as cathepsin C (CTSC), tetratricopeptide repeat domain 5 (TTC5), and upregulated angiogenesis promoting genes such as R-spondin 3 (RSPO3) (Zheng et al., 2016).

#### Anti-cardiac Fibrosis and Anti-cardiac Hypertrophy

Cardiac fibrosis is a common reaction of the heart to many kinds of injuries and is the key pathological process in various CVDs (Mohammed et al., 2015). In cardiac fibrosis, excessive collagen deposition and accumulation of extracellular matrix lead to a decline in myocardial compliance and electrical conduction is also affected, further result in cardiac dysfunction, myocardial hypertrophy, HF, and arrhythmias (Creemers and Pinto, 2011; Donekal et al., 2014). Cardiac hypertrophy is originally an adaptive response of the heart to pathophysiologic stimuli, in an attempt to balance the stress in the ventricular wall and preserve cardiac function. However, persistent hypertrophy caused by heart damage such as hypertension or MI can eventually lead to arrhythmias, dilated cardiomyopathy, and HF, which are the leading cause of sudden death (Burchfield et al., 2013; Xie et al., 2013). Nearly 50% of patients with HF have preserved LVEF, with elevated interstitial myocardial collagen content, interstitial fibrosis and cardiomyocyte hypertrophy as prominent features of tissue remodeling (Borlaug and Paulus, 2011; Shah, 2013). Altogether, cardiac fibrosis and hypertrophy are associated with an increased risk of CVDs.

Masson’s trichrome staining showed that the TAC rats had fibrosis, and collagen deposition was apparent. The mitochondria were swollen, and the ridges were broken in the TAC group. Several studies (Xing et al., 2013; Yang et al., 2017a) indicated that WXKL decreased the accumulation of type III collagen fibers via regulating the calcium/calmodulin dependent kinase II (CaMKII) signaling pathway. In addition, many of the cardiac muscle cells had been dissolved, and some gaps appeared, after WXKL drug treatment, the myocardial cells showed slight recovery, but there was still a significant change in the morphology of mitochondria. Moreover, the myocardial cells from TAC/MI rats usually presented abnormal electrophysiological activity, for instance, the action potential duration at 90% repolarization (APD_90_) was significantly prolonged or abnormalities of calcium channel. WXKL-treated group thus showed a shortened APD_90_, decreased the incidences of early afterdepolarizations (EAD) and delayed afterdepolarizations (DADs) (Xing et al., 2013; Yang et al., 2017a). In TAC-induced HF rabbits model, WXKL could promote the mRNA and protein expression of sarcoplasmic reticulum Ca^2+^ ATPase (SERCA2a) (Lian et al., 2014), the pathophysiology of cardiac hypertrophy (Karmazyn et al., 2008; Stansfield et al., 2014; Yano and Okuda, 2014) and the function of WXKL were shown in **Figure 2D**.

Gap junction channels represent the best-known intercellular communication in the cardiovascular system for maintenance of the normal cardiac rhythm, regulation of vascular tone and endothelial function as well as metabolic interchange between the cells (Pieperhoff and Franke, 2007). In the normal adult heart, there exists three main isoforms: Connexin 40 (Cx40), Connexin 43 (Cx43), and Connexin 45 (Cx45), Cx43 is the most abundant and is expressed in atrial and ventricular myocytes (Severs et al., 2008). Previous studies exhibited that Cx43 expression is down-regulated when cardiac hypertrophy occurs (Yang et al., 2012; Wu et al., 2017a,c; Long et al., 2017), further leading to arrhythmia. WXKL protected the ultrastructure of the gap junctions and raised the phosphorylated Cx43 (Cx43-p)/non-phosphorylated Cx43 (Cx43-np) ratio (Wu et al., 2017a,c) and Cx43 mRNA (Yang et al., 2012; Long et al., 2017). Besides, WXKL enabled to significantly enhance the VF threshold (VFT) and reduced Q-T dispersion (Long et al., 2017; Wu et al., 2017a) to suppress arrhythmia. At the level of single cardiomyocytes, hypertrophy is simply defined as an increase in the cardiomyocyte size. WXKL also shortened the Ang II/NE-induced H9C2 cells length and width (Yang et al., 2012; Ren et al., 2016). In addition, WXKL could reduce the expression of the β-catenin protein and c-myc, involving in the Wnt/β-catenin signaling pathway (Wang et al., 2011a).

**FIGURE 3:**
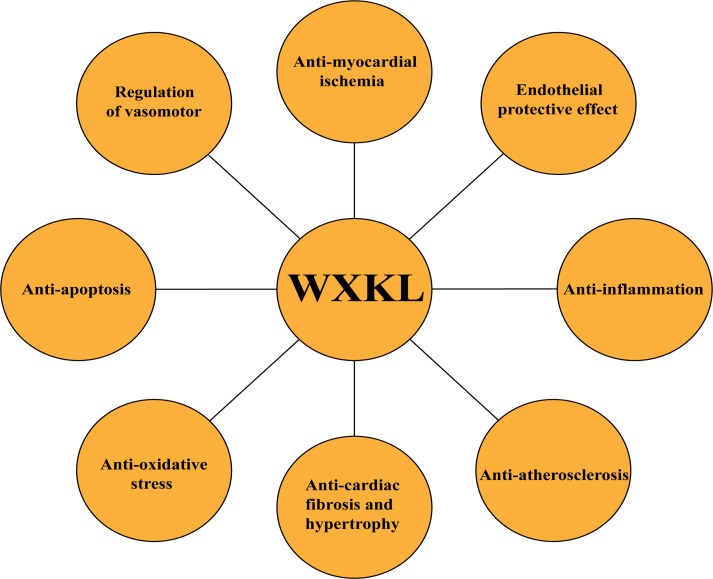
Summary of cardioprotective functions of WXKL.

As stated above, WXKL exerted its cardioprotective effects through inhibiting inflammatory reaction, reducing oxidative stress, regulating vasomotor disorders, decreasing cell apoptosis, and protection against endothelial injure, atherosclerosis, myocardial ischemia, cardiac fibrosis, and cardiac hypertrophy, as shown in **Figures 2A–E**, **3**. In addition, WXKL could raise the serum globulin and serum lysozyme, reduce the serum myocardial enzymes, such as CK isoenzyme (CK-MB), lactate dehydrogenase (LDH), aspartate aminotransferase (AST) (Jin et al., 2006; Zhang et al., 2012), and WXKL also increased the levels of miR-1 and miR-133 (Wu et al., 2017a,b).

### The Antiarrhythmic Effects of WXKL

Several articles exhibited that WXKL played a great role in arrhythmia. In a model of isolated guinea pig hearts, rapid perfusion of quinidine altered the HR and prolonged the Q-T interval, pretreatment with WXKL significantly shortened the QRS and Q-T intervals (Xue et al., 2013; Wang et al., 2017). Besides, WXKL reduced the incidence of VF and the number of ventricular tachycardia (VT) episodes, improved the severity of arrhythmias (Jin et al., 2006; Liang et al., 2012; Liu et al., 2012; Guo et al., 2013; Minoura et al., 2013; Wang et al., 2013). HF was produced by combined aortic insufficiency and abdominal aortic constriction, WXKL could prevent arrhythmias via shortening sinoatrial conduction time (SACT) in HF rabbits (Liu et al., 2011). WXKL also enabled to decreased the maximal diastolic potential (MDP), amplitude of action potential (APA), rate of pacemaker firing (PRF), velocity of diastolic depolarization (VDD), maximal rate of depolarization (Vmax), and so forth (Xu et al., 2010). In addition, persistent AF was inducible in 100% atria pre-treated with Acetylcholine, WXKL prevented the induction of persistent AF in 100% of preparations tested (Wang et al., 2011b) (**Table 2**).

**Table 2:** The antiarrhythmic effect of WXKL.

Experimental models	Targets	Effects	Reference
TAC SD rabbits	EF, FS↑, LVESD↓, LVEDD↓, LVESV↓, LVEDV↓, TDR↓, VFT↑	Anti-arrhythmia and improving cardiac structure	Liu et al., 2012
TAC SD rabbits	SACT↓	Anti-arrhythmia	Liu et al., 2011
MI and depressed SD rats	MAPD_90_↓, Left ventricular-ERP↓, VFT↑	Improving cardiac electrical remodeling	Liang et al., 2012
I/R SPF rats	Incidence of VT+ VF↓, duration of VT +VF↓, CK↓, CK-MB↓, LDH↓, AST↓	Anti-arrhythmia and protecting myocardium	Jin et al., 2006
I/R SPF rats	Na^+^-K^+^-ATPase↑, Ca^2+^ ATPase↑, Mg^2+^ ATPase↑, ST segment elevation_(MAX)_↓	Anti-arrhythmia and improving of myocardial ischemia	Zeng et al., 2006
BL-induced SD rats I/R SD rats	Incidence of VT +VF↓, Duration of arrhythmia↓; Degree of ST segment elevation↓	Inhibiting BL-reduced arrhythmia and protecting myocardium	Guo et al., 2013
ISO-induced SD rats	MDP↓, APA↓, PRF↓, VDD↓, Vmax↓, APD_90_↑	Reducing spontaneous discharge frequency and anti-arrhythmia	Xu et al., 2010
Ischemia-induced rats; Cardiac ventricular myocytes	Incidence of VT +VF↓, Number of episodes of VT+VF↓, number of episodes of VEBs/min↓, arrhythmia score↓, I_CaL_↓, I_to_↓,	Attenuating ischemia-induced ventricular arrhythmias	Wang et al., 2013
Guinea pig hearts	HR↑, QRS↓, Q-T interval↓, Ca_V 1.2_ channel↓	Prevention of quinidine-induced arrhythmia	Wang et al., 2017
Rabbit Purkinje cells	APD↓, EADs↓, DADs↓, TAs↓, I_NaL_↓, I_NaP_↓, I_CaL_↓	Exhibiting antiarrhythmic role via selective inhibition of I_NaL_	Hou et al., 2016
Rats ventricular myocytes	APD↓, I_CaL_↓	Treatment of arrhythmias	Chen et al., 2013a
Rats ventricular myocytes	I_Ca_↓, I–V curve↑	Anti-arrhythmia	Wang et al., 2011b
Rats ventricular myocytes	I_to_↓, I–V curve↓	Anti-arrhythmia	Wang et al., 2010
Rabbit ventricular myocytes	I_NaL_↓, I_NCX_↓, I_CaL_↓, APD↓	Inhibiting hypoxia-reoxygenation induced tachycardia	Luo et al., 2017
Rabbit ventricular myocytes	I_NaL_↓, EADs↓, DADs↓, Q-T interval↓	Inhibiting ventricular arrhythmias	Xue et al., 2013
Rabbits ventricular myocytes	APD_90_↑, TDR↓	Anti-arrhythmia	Cui, 2007
Canine cardiomyocytes	P2R↓, VT↓, VF↓, NMI↓, J Wave Area↓, I_to_↓, I_Ca_↓	Suppressed arrhythmogenesis of Brugada syndrome	Minoura et al., 2013
Canine atrial and ventricular myocytes, HEK293 Cells	Atrial-selective Na^+^ channels↓	Suppression of atrial fibrillation	Hu et al., 2016
Canine arterially perfused right atrial preparations with a rim of right ventricular tissue; HEK293 cells	APD_90_↓, Atrial-selective ERP↑, I_Na_↓	Suppressed atrial fibrillation	Burashnikov et al., 2012
Ouabain-perfused ventricular myocytes	APD_90_↑, EAD↓, TA↓	Anti-arrhythmia	Chen et al., 2010
Xenopus oocyte	HCN2 instantaneous currency↑	Regulation of HCN2 channel	Li et al., 2011

*I_*NCX*_, Na^+^–Ca^*2*+^ exchange current; I_*NaP*_, peak sodium current; GP, ganglionic plexi; VEB, ventricular ectopic beats; I_*to*_, transient outward potassium current; P2R, phase 2 re-entry; NMI, notch magnitude index; ERP, effective refractory period; BL, barium chloride; MAP, monophasic action potential; VFT, ventricular fibrillation threshold; I–V, current–voltage; TDR, transmural repolarization dispersion; HCN2, hyperpolarization-activated cyclic nucleotide-gated cation channels-2*.

**FIGURE 4:**
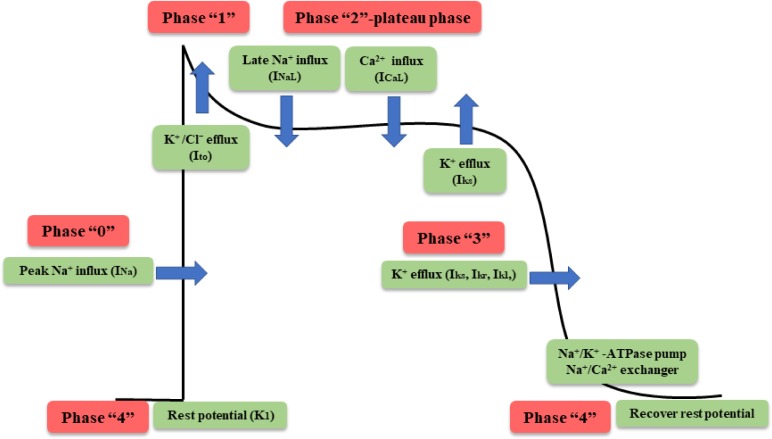
Phases of cardiac action potential in nonpacemaker cells.

The generation of myocardial cell action potentials is the basis of cardiac electrical activity, which includes five phases (from 0 to 4) (Tse, 2016). Depolarization from the sinoatrial node brings the membrane potential to the threshold, opening the voltage-gated sodium channels and giving rise to the peak current of sodium (I_Na_) and the rapid upstroke (phase 0) of the cardiac action potential. Inactivation of the sodium channel and activation of transient outward potassium current (I_to_) are the predominant contributors to the partial membrane repolarization in the first phase. Phase 2, the videlicet plateau, is a long phase due to the delicate balance between the inward currents (L-type calcium current, I_CaL_ and late sodium current, I_NaL_) and the outward currents (slow delayed rectifier K+ channels, I_ks_). As the inward currents (I_CaL_) become inactivated, the outward currents (I_ks_; rapid delayed rectifier K+ channels, I_kr_ and inwardly rectifier K^+^ channels I_k1_) predominate, causing further repolarization and bringing the membrane potential toward the potassium equilibrium potential (phase 3). Then, the membrane potential returns to its resting potential after full repolarization during phase 4, which depends on numerous components (Na^+^/K^+^ – ATPase pump, the Na^+^/Ca^2+^ exchanger) to recover the normal concentration gradient of the myocardial cell membrane (**Figure 4**). WXKL inhibits various cardiac ion channels with different potencies acting mainly on peak and late I_Na_, with some effect on I_to_ and I_CaL_ channels.

#### Role of WXKL in Sodium Channels

Sodium channels are widely distributed in myocardial cells in mammals and mediate the excitability and conduction of the heart. There are main two sub-types of sodium currents, one is the I_Na_, the other is the I_NaL_. I_Na_ is primarily responsible for depolarization (phase 0) in myocardial action potentials while I_NaL_ maintains balance in the plateau as a kind of inward current. WXKL produces atrial-selective depression of I_Na_-dependent parameters in canine isolated coronary perfused preparations and was effective in reducing I_Na_ and shifting steady-state inactivation to more negative potentials in HEK293 cells stably expressing SCN5A (Burashnikov et al., 2012). Moreover, WXKL could inhibit I_Na_ more preferentially in the atria and lowered resting membrane potential, resulting in post-repolarization refractoriness in atrial myocytes, and therefore suppressed AF via an anti-re-entrant mechanism (Hu et al., 2016).

**FIGURE 5:**
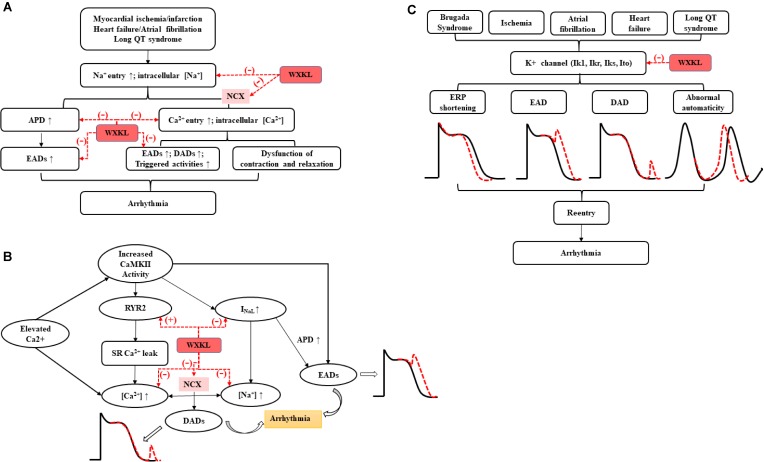
The antiarrhythmic effects of WXKL on **(A)** sodium channels, **(B)** calcium channels, and **(C)** potassium channels.

More recent evidence suggests the I_NaL_, which lasts hundreds of milliseconds after a depolarizing pulse, may delay repolarization, prolong APD, alter intracellular sodium and calcium homeostasis, and lead to EAD/DAD and a series of triggered activities, potentially predisposing to arrhythmias and sudden cardiac death (Shryock et al., 2013; Zaza and Rocchetti, 2013; Horvath and Bers, 2014; Remme and Wilde, 2014). WXKL exhibited antiarrhythmic properties via selective inhibition of I_NaL_ (Xue et al., 2013; Hou et al., 2016). In addition, WXKL attenuated intracellular Ca^2+^ overload induced by hypoxia-reoxygenation in ventricular myocytes via inhibiting I_NaL_ and finally prevented the occurrence of arrhythmia (Luo et al., 2017). It is all known that cardiac Purkinje cells (PCs) played a crucial role in the propagation of impulse from atrioventricular node to ventricular muscle and may initiate a variety of ventricular arrhythmias (Huang et al., 2014). WXKL effectively abbreviated the APD of PCs in a dose- and rate-dependent manner by recording ion currents through whole-cell patch clamp technique, and inhibited ISO-induced EADs, DADs, and TAs by selective inhibition of I_NaL_ (Hou et al., 2016). The mechanism of sodium channel regulating arrhythmia (Remme and Wilde, 2014) and the effects of WXKL were shown in **Figure 5A**.

#### Role of WXKL in Calcium Channels

There are three types of calcium channels in myocardial cells, i.e., B-type, L-type, and T-type (Rosenberg et al., 1988). L-type and T-type calcium channels plays major roles in myocardial electrophysiological activity. The activated L-type calcium channel is capable of generating a slow I_CaL_, which is the ionic basis of ventricular cardiac action potentials during phase 2 (Yamaoka and Kameyama, 2003). T-type calcium channels mainly exist in cardiac autonomic cells, such as sinoatrial node cells, and are activated to affect the pacemaker activity of the heart through a slow inward calcium current (I_CaT_) (Ono and Iijima, 2010).

Wenxin Keli could attenuate intracellular Ca^2+^ overload in ventricular myocytes by suppressing the I_CaL_ (Luo et al., 2017). Additionally, WXKL inhibits I_CaL_ decelerating the activation process and delaying recovery from inactivation without changing the inactivation process (Wang et al., 2013). In the normal and hypertrophied myocytes, WXKL decreased the I_CaL_ by accelerating the inactivation of the channels and delaying the recovery time from inactivation, which ultimately resulted in the treatment of arrhythmias (Chen et al., 2013a). WXKL also inhibited the current density of I_CaL_ in a concentration-dependent manner, decreased rates of the peak I_CaL_ and shifted up the current–voltage curve (Wang et al., 2011b). Besides, *Nardostachys chinensis Batal (Gan Song)*, one of the WK components, dose-dependently blocked its predicted target Ca_V 1.2_ channel in an electrophysiological assay (Wang et al., 2017).

The past decade has seen the emergence of CaMKII as a critical regulator in many cardiac pathologies, especially arrhythmia. Altered L-type Ca^2+^ channel gating due to CaMKII hyper-activity could elicit EADs by increasing I_CaL_. Na_V 1.5_ phosphorylation increases I_NaL_ and may result in non-equilibrium reactivation of I_Na_, both of which can prolong APD and trigger EADs. Besides, Increased CaMKII activity in disease has been linked to elicit gain-of-function effects at I_CaL_, thereby increasing Ca^2+^ influx and leading to Ca^2+^ overload. CaMKII also could contribute to Na^+^-induced Ca^2+^-overload through NCX. Moreover, CaMKII directly phosphorylates RyR2, which has been shown to promote spontaneous Ca^2+^ release. Both of above processes can further trigger DADs, resulting in the cardiac arrhythmia (Asakura et al., 2014; Vincent et al., 2014). Many studies demonstrated the functions of WXKL on arrhythmia via regulating calcium channels, reducing the I_CaL_, preventing the occurring of EAD and DAD via acting on CaMKII signaling pathway (Chen et al., 2013a; Xing et al., 2013; Xue et al., 2013; Hou et al., 2016; Luo et al., 2017; Yang et al., 2017a) (**Figure 5B**).

#### Role of WXKL in Potassium Channels

There are four types of potassium channels in myocardial cells, i.e., I_to_, I_k1_, I_kr_, and I_ks_. The role of I_k1_, I_kr_, and I_ks_ are to influence the resting potential of the myocardial cell membrane and repolarization (phases 2 and 3) in myocardial cells (Grandi et al., 2017). I_to_, the main current responsible for the early rapid repolarization (phase 1) in fast response cells, has been proven to exist extensively in myocardial cells, especially in atrial and ventricular myocytes in mammals. It has a significant effect on the shape and the duration of the cardiac action potential (Barry et al., 1998). In addition, the I_to_ is characterized by a transmural gradient in current density across the ventricular myocardium that leads to significant differences in cardiac action potentials between the endocardium and the epicardium (Perrin et al., 2014). This distribution gives rise to repolarization heterogeneity and is probably responsible for the main pathogenesis of VT and VF. WXKL mainly acted on potassium ion channels and regulated the abnormal production of I_to_ (Wang et al., 2010, 2013; Minoura et al., 2013).

Brugada syndrome (BrS) is an inherited cardiac disorder characterized by a coved-type ST-segment elevation in the right precordial leads and increased risk of associated with a high incidence of sudden death due to the development of life-threatening polymorphic VT and VF (Sieira and Brugada, 2017). The present study showed the effect of WXKL to suppress the electrocardiographic and arrhythmic manifestations of BrS in a coronary-perfused canine RV wedge model of the syndrome. Its function to inhibit phase 2 re-entry and VT/VF involved the prevention of I_to_ as well as an indirect adrenergic stimulation via a tyramine-like effect (Minoura et al., 2013). In addition, the present study demonstrated that WXKL enabled to reduce ischemia-induced ventricular arrhythmias in rats by inhibiting I_to_ in a concentration-dependent manner (Wang et al., 2013). WXKL also suppressed the current density of I_to_, decreased rates of the peak I_to_ and shifted down the current–voltage curve in single ventricular myocytes (Wang et al., 2011b). The functional roles of potassium currents in the arrhythmias (Chiamvimonvat et al., 2017) and intervention of WXKL were shown in **Figure 5C**.

#### Role of WXKL in HCN Channels

Hyperpolarization-activated, cyclic nucleotide-gated (HCN) channels belong to the superfamily of voltage-gated pore-loop cation channels, including HCN1–HCN4 isoforms (Santoro and Tibbs, 1999). Human genetic and animal studies have highlighted the involvement of HCN family of channels in the heart where they are responsible for the pacemaker current (I_f_) current in the sinoatrial node (Herrmann et al., 2012). In the ventricular tachypacing-induced CHF dogs, HCN2 and HCN4 expression was higher in sinoatrial node than the right atrium. CHF significantly reduced sinus node HCN expression at both protein and mRNA levels (Zicha et al., 2005). WXKL enhanced the amplitude of instantaneous current of HCN2 in a concentration-dependent manner, slowed channel activation and deactivation processes (Li et al., 2011).

## Discussion

In the past two decades, a breakthrough has been achieved in the pharmacology of WXKL. The knowledge of WXKL offers a new chance for the prevention and treatment of CAD, mainly reflecting in two aspects: cardioprotective effects and anti-arrhythmias. WXKL exerted its cardioprotective properties by inhibiting inflammatory reaction, decreasing oxidative stress, regulating vasomotor disorders, lowering cell apoptosis, and protection against endothelial injure, myocardial ischemia, cardiac fibrosis, and cardiac hypertrophy. Besides, WXKL could effectively shorten the QRS and Q-T intervals, decreased the incidence of VF and the number of VT episodes, improved the severity of arrhythmias by regulating various ion channels with different potencies, mainly comprising peak and late I_Na_, I_to_, I_CaL_, and I_f_. WXKL also enabled to decreased the MDP, APA, PRF, VDD, Vmax, and so forth.

The pharmacological effects of WXKL were closely related to its components. As we all know, WXKL is a Chinese herb extract that includes five main components: *Codonopsis Radix (Dang Shen), Polygonati Rhizoma (Huang Jing), Notoginseng Radix Et Rhizoma (San Qi), Ambrum (Hu Po), and Nardostachyos Radix Et Rhizoma (Gan Song).* Developed in various forms over the past 2,000 years, the main components of WXKL were used by millions today for the treatment of a variety of CVDs. *Codonopsis Radix (Dang Shen)*, a TCM herb, has been used in clinical applications for hundreds of years. Several studies have focused on its immune-enhancing properties through acting on the regulation of the lymphocytes, cytokines, antibody level, and the neuroendocrine-immune network (Zhao et al., 2013). *Notoginseng Radix Et Rhizoma (San Qi)*, a valuable herb in TCM with obvious efficacy and favorable safety, played a crucial role in CADs and was increasingly recognized clinically. Many experimental and clinical studies have demonstrated that *Notoginseng Radix Et Rhizoma (San Qi)* could regulate lipid metabolism, reduce the inflammatory reaction and myocardial damage, improve the energy metabolism of myocardial cells, inhibit ischemia-induced cardiomyocyte apoptosis in acute MI rats and so forth (Duan et al., 2017). *Nardostachyos Radix Et Rhizoma (Gan Song)*, the rhizomes and roots of Nardostachys jatamansi DC, could rectify qi, relieve pain, resolve constraint and fortify the spleen. In addition, *Nardostachyos Radix Et Rhizoma (Gan Song)* exhibited the cardioprotective effects via inhibiting myocardial apoptosis, inflammation, oxidative stress and showed a great promise as a novel option for arrhythmia by regulating several ion channels (I_k_, I_k1_, I_Na_, I_CaL_, and I_to_) (Li et al., 2017b).

**FIGURE 6:**
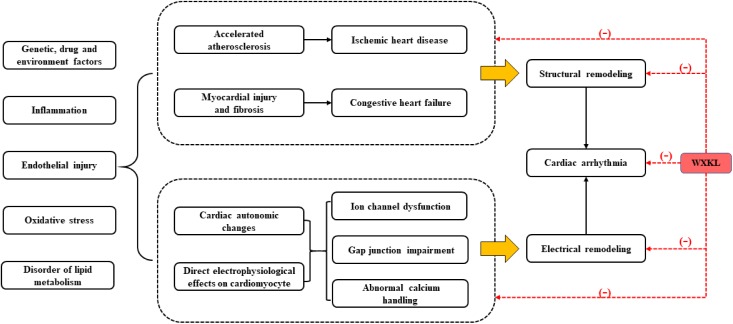
The comprehensive effects of WXKL in CVDs.

Genetic, drug and environment factors, oxidative stress, inflammatory cytokines, disorder of lipid metabolism, endothelial injury, all of which cannot only lead to atherosclerosis, myocardial injury and fibrosis, but also result in cardiac autonomic changes and electrophysiological abnormalities of cardiomyocytes, eventually causing cardiac remodeling. Such cardiac remodeling involves complex structural remodeling and electrical remodeling, which is more commonly seen in the myocardial injury, especially in coronary artery disease. Cardiac remodeling is often used as an adaptive response to functional or structural stress, which initially compensates and maintains cardiac function, but it may then turn into a non-adaptive change, causing progressive pump failure and/or malignant arrhythmia (Lazzerini et al., 2017) (**Figure 6**). WXKL exhibited multiple protective effects on myocardium and regulation functions on ion channels, these two properties were complementary and beneficial to each other. In brief, WXKL comprehensively treats and regulates CVDs.

A large number of studies have shown that lipid metabolism disorders play an important role in the pathogenesis of atherosclerosis and cardiac death in CVDs, specifically increased total cholesterol (TC), triglycerides (TG), LDL-C, and ox-LDL (Charland and Stanek, 2014). In clinical practice, WXKL could regulate lipid metabolism by reducing TC, TG, LDL, and raising HDL and the level of apolipoprotein A (ApoA)/apolipoprotein B (ApoB) in patients with incidental atrial premature (Li, 2018). *Panax notoginseng (San Qi)*, one of main components of WXKL, could decrease cholesterol ester in foam cells by up-regulation of ATP-binding cassette transporter A1 (ABCA1) and ATP-binding cassette transporter G1 (ABCG1) (Jia et al., 2010; Fan et al., 2012). However, there is no report of WXKL to regulate dyslipidemia in the current experimental study. The effect of WXKL on regulating lipid metabolism remains to be further studied.

Nowadays, anti-arrhythmic drugs were divided into four categories according to the Vaughan-Williams classification scheme (Vaughan-Williams, 1977). Among them, the third anti-arrhythmic drugs (mainly inhibiting I_k+_) have the functions of prolonging ADP. Several studied presented that WXKL may own the similar effect (Cui, 2007; Chen et al., 2010; Xu et al., 2010). However, compared with I_k+_, some experiment studies exhibited that I_Na-L_ and I_Ca_ have opposite effects on APD, causing the prolongation of APD and the triggering of EADs/DADs. WXKL has played an important role in shortening APD_90_ and decreasing the incidences of EADs/DADs (Chen et al., 2013a; Xue et al., 2013; Hou et al., 2016; Luo et al., 2017). WXKL could inhibit above currents simultaneously, thus, WXKL has a double-sided effect on APD, the anti-arrhythmic mechanism will depend on the cell type, as well as the degree of contribution of these currents in repolarization at any given time.

## Limitations and Perspectives

There were some limitations in this review. Firstly, taking nuclear factor-κB, PLC, protein kinase B for instance, many mechanisms of protecting myocardium with WXKL are still not clear. Secondly, the studies mainly involved cardiac working cells (atrial and ventricular myocytes), the other autonomous cells, such as sinoatrial node cells and Purkinje cells, were lack of more research, resulting in the insufficient evidence on the mechanism of WXKL. Then, no studies have reported the effects of WXKL on I_CaT_, I_ks_, or I_kr_. And current studies showed that WXKL enabled to inhibit the tachyarrhythmia, however, as for the bradyarrhythmia, whether WXKL is an appropriate medicine remains unclear. In view of above deficiencies, more studies need to be further explored.

## Conclusion

Wenxin Keli has been observed to have multiple positive functions in CVDs, including myocardial protection and anti-arrhythmia. WXKL played its cardioprotective roles by inhibiting inflammatory reaction, decreasing oxidative stress, regulating vasomotor disorders, lowering cell apoptosis, and protection against endothelial injure, myocardial ischemia, cardiac fibrosis, and cardiac hypertrophy. Besides, WXKL could effectively shorten the QRS and Q-T intervals, decreased the incidence of VF and the number of VT episodes, improved the severity of arrhythmias by regulating various ion channels with different potencies, mainly comprising peak and late I_Na_, I_to_, I_CaL_, and I_f_.

## Author Contributions

HS, ML, and YL provided guidelines for this review. ML, GT, and YS wrote the main manuscript and prepared the **Figures 1**–**6**. SL, CL, SC, RQ, and XZ reviewed the literature available on this topic and prepared the **Tables 1**, **2**.

## Conflict of Interest Statement

The authors declare that the research was conducted in the absence of any commercial or financial relationships that could be construed as a potential conflict of interest. The reviewer RZ and handling Editor declared their shared affiliation.
