# Supplementary material for: Therapeutic Effects of Wenxin Keli in Cardiovascular Diseases: An Experimental and Mechanism Overview
Source: Front Pharmacol. 2018 Sep 5;9:1005. doi: 10.3389/fphar.2018.01005 (PMC6134428; doi:10.3389/fphar.2018.01005)
Supplement: Supplementary file 1 [file Table_1.DOCX]

Supplementary Material 1

1 The description of Wenxin Keli


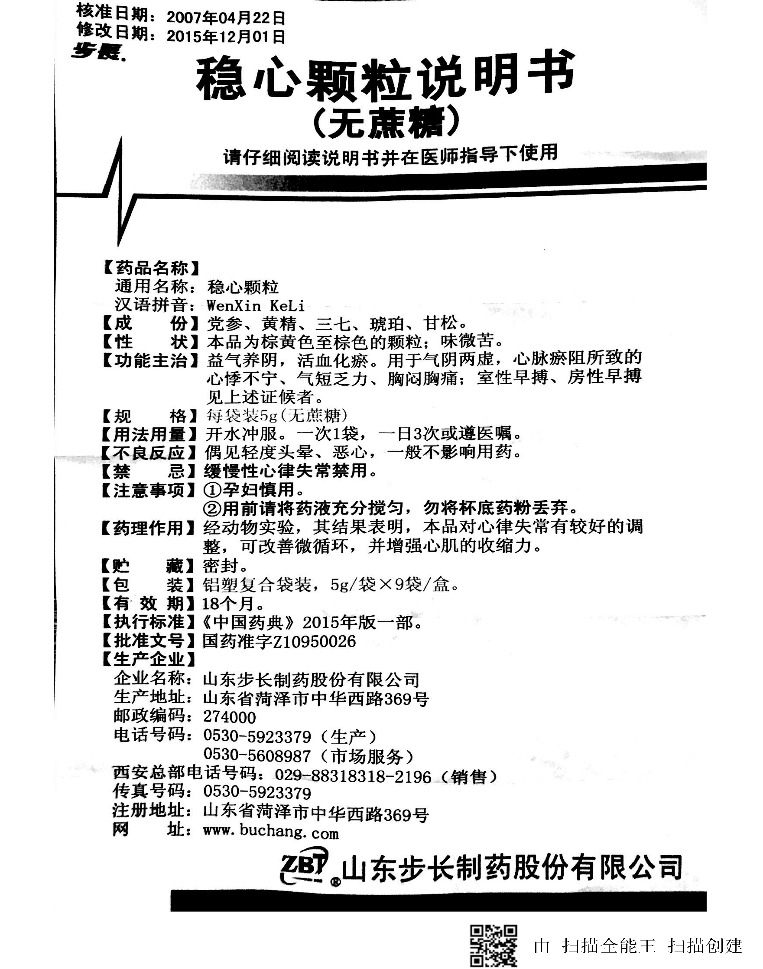


2 The detailed information of Wenxin Keli

2.1 Ingredients: *Codonopsis Radix (Dang Shen), Polygonati Rhizoma (Huang Jing), Notoginseng Radix Et Rhizoma (San Qi),* *Ambrum (Hu Po) and Nardostachyos Radix Et Rhizoma (Gan Song).*

2.2 Preparation procedure:

2.2.1 Ambrum (Hu Po) was pulverized into fine powder, and *Nardostachyos Radix Et Rhizoma (Gan Song)* extracted volatile oil.

2.2.2 *Notoginseng Radix Et Rhizoma (San Qi)* was pulverized into coarse powder, extracted twice with 80% ethanol under reflux for 2 hours and concentrated to a suitable clearing paste. The dregs were boiled twice with water, the first time was 2 hours, the second time was 1.5 hours, and the decoction was combined.

2.2.3 *Codonopsis Radix (Dang Shen)* and *Polygonati Rhizoma (Huang Jing)* added water to cook twice, the first time was 2 hours, the second time was 1.5 hours, and the decoction was combined with the above decoction (the dregs of *San Qi*) and filtered.

2.2.4 The above filtrate was concentrated to a clear paste and had a relative density of 1.20 to 1.30 (60 °C), ethanol was added to make the alcohol content up to 65 %, stirred, and stood for 24 hours, filtered, and the filtrate was concentrated into a suitable thick paste, and combined with clearing paste of *Notoginseng Radix Et Rhizoma (San Qi)*.

2.2.5 Adding the above fine powder of Ambrum (Hu Po), 518g of sucrose, 100g of beta cyclodextrin, 6.5g of aspartame, and a proper amount of dextrin, granulating, drying, spraying volatile oil of *Nardostachyos Radix Et Rhizoma (Gan Song)*, mixing, and making 1000g of granules.

2.2.6 Or add the above Ambrum (Hu Po) fine powder, aspartame, beta cyclodextrin and soluble starch, granulating, drying, spraying volatile oil of *Nardostachyos Radix Et Rhizoma (Gan Song)*, mixing, and making 556g of granules (without sucrose).

2.3 Clinical dosage: one bag each time, three times a day.

2.4 Size: (1) 9g per bag (2) 5g per bag (no sucrose).
